# Transcriptome Analysis of the Role of GlnD/GlnBK in Nitrogen Stress Adaptation by *Sinorhizobium meliloti* Rm1021

**DOI:** 10.1371/journal.pone.0058028

**Published:** 2013-03-13

**Authors:** Svetlana N. Yurgel, Jennifer Rice, Michael L. Kahn

**Affiliations:** 1 Institute of Biological Chemistry, Washington State University, Pullman, Washington, United States of America; 2 School of Molecular Biosciences, Washington State University, Pullman, Washington, United States of America; Centre National de la Recherche Scientifique, Aix-Marseille Université, France

## Abstract

Transcriptional changes in the nitrogen stress response (NSR) of wild type *S. meliloti* Rm1021, and isogenic strains missing both PII proteins, GlnB and GlnK, or carrying a Δ*glnD*-sm2 mutation were analyzed using whole-genome microarrays. This approach allowed us to identify a number of new genes involved in the NSR and showed that the response of these bacteria to nitrogen stress overlaps with other stress responses, including induction of the *fixK2* transcriptional activator and genes that are part of the phosphate stress response. Our data also show that GlnD and GlnBK proteins may regulate many genes that are not part of the NSR. Analysis of transcriptome profiles of the Rm1021 Δ*glnD*-sm2 strain allowed us to identify several genes that appear to be regulated by GlnD without the participation of the PII proteins.

## Introduction

Nitrogen is an essential chemical element for all living organisms. It is an irreplaceable component of proteins, DNA and RNA – the building blocks in every living cell on Earth. In the form of dinitrogen (N_2_) it constitutes 78% of the atmosphere. However, because of the inert nature of N_2_, nitrogen availability is a major factor limiting plant and bacterial development. To survive in an environment with fluctuating nitrogen availability, bacteria developed physiological mechanisms for its efficient acquisition and utilization. The bacterial **N**itrogen **S**tress **R**esponse (NSR) regulatory cascade is well defined, especially in enteric bacteria [1], [2]. The uridylyltransferase and uridylyl cleavage enzyme GlnD is a primary sensor that activates the NSR in nitrogen-limited cells by uridylylating PII proteins (usually GlnB and GlnK). Modification of the PII proteins changes their ability to modulate the activity of numerous targets, such as the AmtB ammonium transporter and the glutamine synthetase (GS)-adenylyl-transferase, GlnE. The activity of a two component regulatory system, NtrBC, is also controlled by PII protein modification, resulting in NtrC phosphorylation and activation of nitrogen catabolism and assimilation under nitrogen limitation [2]. In *E. coli*, communication between GlnD and the NSR is mediated by the PII proteins; in a mutant lacking both GlnB and GlnK, the NSR phenotype does not depend on presence of GlnD [3].

Some bacteria have the ability to utilize atmospheric N_2_ by reducing (fixing) it to ammonia. Free-living diazotrophs, such as *Azotobacter* or *Klebsiella,* only employ nitrogen fixation when other sources of nitrogen are not available and induce nitrogen fixation as part of the NSR [4]. On the other hand, symbiotic diazotrophs like *Sinorhizobium meliloti* fix nitrogen in association with *Medicago* plants [5]. The symbiotic rhizobia activate N_2_ fixation while at the same time limiting ammonia assimilation into amino acids [6], indicating that symbiotic nitrogen fixation is not coordinated with the NSR. **G**lutamine **S**ynthetase (GS) is a major enzyme involved in ammonia assimilation. *S. meliloti* has two primary glutamine synthetases (GSI and GSII), and a third, GSIII, which is expressed only when GSI and GSII are missing [7]. Similar to *Rhizobium leguminosarum* [8], *S. meliloti* Rm1021 *glnB* mutants highly overproduce GSII, implying that the absence of GlnB results in NtrC phosphorylation [9], which is characteristic of a strongly induced NSR. Moreover, mutations in the GlnD protein impaired the ability of *S. meliloti* and *R. leguminosarum* to produce GSII under nitrogen limitation, suggesting that GlnD was required for NtrC phosphorylation, probably through GlnB uridylylation [10].

The deletion of the N-terminal uridylyltransferase domain in GlnD (strain Rm1021Δ*glnD*-sm2) causes misregulation of nitrogen exchange in the *S. meliloti*-alfalfa association, resulting in a symbiosis where the fixed nitrogen is not available for plant use [11]. This indicated the importance of the GlnD protein in the ability of *S. meliloti* to establish effective symbiosis with the host plant. However, an isogenic *S. meliloti* strain missing both GlnB and GlnK proteins could establish an effective symbiotic association that provided the host-plant with nitrogen sufficient for growth, while combining a *glnD* mutation with the *glnB*/*glnK* deletions led to a Fix+Eff symbiotic phenotype similar to the Rm1021Δ*glnD*-sm2 symbiotic phenotype. Furthermore, the triple *glnD/glnB*/*glnK* mutant had more severe growth defects than the *glnBglnK* mutant [9], [12]. This suggested GlnD has a role in *S. meliloti* free-living growth and symbiotic nitrogen exchange that does not depend on the PII proteins.

In this study we employed a whole-genome *S. meliloti* microarray to analyze the transcriptional response to nitrogen starvation of wild type Rm1021 and of mutant strains missing both PII proteins or carrying the Δ*glnD*-sm2 mutation. This approach allowed us to identify a number of new genes involved in the NSR and to show that the expression of many genes that are not affected in a major way by nitrogen stress are affected by truncation of GlnD and/or the GlnB and GlnK deletions. The rhizobial response to nitrogen stress also appears to be integrated with other stress responses, including the phosphate stress response and genes activated by the FixK2 transcriptional activator.

## Materials and Methods

### Bacterial Strains and Growth Conditions

The strains Rm1021 [13], Rm1021Δ*glnD-sm2* [11], and Rm1021Δ*glnB*Δ*glnK* [9] were grown with constant agitation (250 rpm) at 30°C in Minimal Mannitol (MM) medium [14] with 0.05% ammonium chloride (MM-NH_4_) or 0.02% sodium glutamate (MM-Glu) as nitrogen sources. At least three biological replicates, grown to mid-log phase (0.5–0.6 OD_600_), were used for each data set.

### Quantitative Analysis of High-molecular-weight Exopolysaccharide (HMW EPS) Production

EPS analysis was done as described by Marroqui *et al*. [15] with modifications. *S. meliloti* strains were first grown for 48 h in MM-NH_4_ medium at 30°C with shaking to late log phase. 2 ml of the culture was used to inoculate 250 ml of MM-NH_4_ or MM-Glu. The cells were grown at 30°C with shaking. 50 mL from the culture was harvested at 3, 5, 7, and 10 days and centrifuged for an hour at 8000 rpm. The supernatants were combined with 0.88 g NaCl and 125 ml 100% ethanol and incubated at 4C overnight. The next morning, polysaccharides were removed from the ethanol with a glass rod and transferred to a pre-weighed petri plate and dried overnight at 40°C.

### RNA Isolation, cDNA Synthesis, Labeling, and Hybridization

RNA was isolated according to the protocol published by *Barnett et al.* [16]. Total RNA was prepared using Qiagen RNeasy bacterial RNA purification kits (Qiagen, Chatsworth, CA) according to the manufacturer’s protocol. DNA contamination of the RNA was checked by PCR according to the protocol published by *Barnett et al.* [16]. RNA quality was analyzed on a 1% agarose gel, and RNA quantity was measured using a nanoDrop ND-1000 spectrophotometer (Thermo Scientific, Wilmington, U.S.A.).

Total RNA was processed according to the Affymetrix User Guide, Prokaryotic Sample and Array Processing. Briefly, first-strand cDNA was synthesized using random primers and SuperScript II Reverse Transcriptase (Invitrogen Life Technologies, Foster City, CA). RNA was degraded using NaOH, the sample was neutralized with HCl and the resulting single stranded cDNA was purified on a Qiagen MinElute PCR purification column (Qiagen, Chatsworth, CA). Single stranded cDNA was fragmented using DNAse I (Amersham Biosciences) and then end labeled with biotin using the GeneChip DNA Labeling Reagent (Affymetrix, Santa Clara, CA) and terminal deoxynucleotidyl transferase (Promega, Fitchburg, WI). Prepared targets were hybridized at 48°C for 16 hr in the GeneChip Hybridization Oven 640 according to the manufacturer’s recommendations. Hybridized arrays were washed and stained using the GeneChip Fluidics Station 450. The fluidics protocol FlexMidi_euk2v3_450 was used with recommended modifications as well as changing the Wash B Temperature from 50°C to 48°C. Processed arrays were scanned using a GeneChip 3000 7G scanner.

### Data Analysis

Array quality was assessed, and images were quantified using GeneChip Operating Software v1.2 (Affymetrix). The data were viewed and analyzed using Partek Genomic Suite 6.5 beta software (Partek Incorporated, St. Louis, MO). The genes with absolute expression ratio ≥2 (p<0.05) were considered induced or repressed. The list of differentially expressed genes was created using analysis of variance (ANOVA). The microarray data from this study are compiled in the NCBI Gene Expression Omnibus (GEO) database (http://www.ncbi.nlm.nih.gov/geo/) and are accessible through the GEO series accession number GSE43570.

### Quantitative PCR

Relative transcription of selected genes was determined by real–time qRT-PCR. Primers were designed with AutoDimer software [17] to amplify 80- to 250- bp regions of the chosen genes (Table S1). cDNA was synthesized using the High Capacity cDNA Reverse Transcription Kit (Applied Biosystems) according to the manufacturer’s protocol. Gene expression differentials were estimated as the ratios of normalized gene expression using Q-gene software [18]. Relative expression was normalized to the expression value of SMc02641, which was found to be expressed at similar levels under high and low nitrogen conditions in all strains.

## Results and Discussion

### Conditions and Strains Used for Microarray Transcriptome Profiling

To induce nitrogen stress, we grew *S. meliloti* strains Rm1021, Rm1021Δ*glnD-*sm2 and Rm1021Δ*glnB*Δ*glnK* on MM-Glu media (low nitrogen). Nitrogen excess conditions were achieved by growing the cells in MM-NH_4_ media (high nitrogen). Previously we showed the presence of these two nitrogen sources in growth media significantly affected the expression level of GSII and modification state of GSI [11] in *S. meliloti* Rm1021 and we concluded that these media were suitable for creating nitrogen limited (Glu) and nitrogen sufficient (NH_4_) growth conditions. To study the NSR we analyzed the changes in gene expression in the *S. meliloti* wild type strain Rm1021 grown under low or high nitrogen (Table 1, Cluster I and II). To dissect the role of GlnD and PII proteins in NSR we compared the expression profiles of Rm1021Δ*glnD-*sm2 and Rm1021Δ*glnB*Δ*glnK* strains grown under low or high nitrogen (Table 1, Cluster III). Our previous analysis of the function of the GlnD-GlnB/K regulatory cascade suggested an involvement of these proteins in a broader range of physiological functions than the NSR [9], [11]. To identify the potential targets of GlnD or/and GlnB/K proteins, we compared the expression profiles of the mutants to the wild type strain and to each other after each strain was grown under the same nitrogen availability conditions (Table 1, Cluster III).

**Table 1:** Comparison of transcriptome profiles used in the study.

Transcriptome profiling	Notation	Cluster
Rm1021-glu^1^ vs. Rm1021-NH_4_^2^	1021-Glu vs. 1021-NH_4_	Cluster I and II
Rm1021Δ*glnD*-sm2-glu vs. Rm1021Δ*glnD*-sm2-NH_4_	D-Glu vs. D- NH_4_	Cluster III
Rm1021Δ*glnB*Δ*glnK*-glu vs. Rm1021Δ*glnB*Δ*glnK*-NH_4_	BK-Glu vs. BK-NH_4_	Cluster III
Rm1021-glu vs. Rm1021Δ*glnD*-sm2-glu	1021-Glu vs. D-Glu	Cluster III
Rm1021-glu vs. Rm1021Δ*glnB*Δ*glnK*-glu	1021-Glu vs. BK-Glu	Cluster III
Rm1021Δ*glnB*Δ*glnK*-glu vs. Rm1021Δ*glnD*-sm2-glu	BK-Glu vs. D-Glu	Cluster III
Rm1021-NH_4_ vs. Rm1021Δ*glnD*-sm2-NH_4_	1021-NH_4_ vs. D-NH_4_	Cluster III
Rm1021-NH_4_ vs. Rm1021Δ*glnB*Δ*glnK*-NH_4_	1021-NH_4_ vs. BK-NH_4_	Cluster III
Rm1021Δ*glnD*-sm2-NH_4_ vs. Rm1021Δ*glnB*Δ*glnK*-NH_4_	BK-NH_4_ vs. D-NH_4_	Cluster III

The cells were grown in MM media supplemented with: 1–0.02% sodium glutamate as nitrogen source and 2–0.05% ammonium chloride as nitrogen source.

By comparing transcriptome profiles of the *S. meliloti* wild type strain and the regulatory mutants, grown under low or high nitrogen, we identified 609 genes whose expression was significantly affected by nitrogen stress or by the mutations in GlnD or the PII proteins. The initial criteria used to identify a significant change in gene expression were a 2.0-fold change between conditions with a *P* value of <0.05, although these criteria were relaxed as described below in order to include operons as coordinately expressed genes. A relatively small fraction of the genes were directly affected by nitrogen stress. When Rm1021 cells grown on glutamate were compared to cells grown on ammonium, only 52 of the 609 genes (8.5%) were downregulated and 78 genes (12.8%) were upregulated. Since many NSR genes are involved in catabolism of specific compounds that are often inducers of the genes, there are likely to be NSR genes not identified here that are co-regulated by the catabolic substrate.

**Figure 1:**
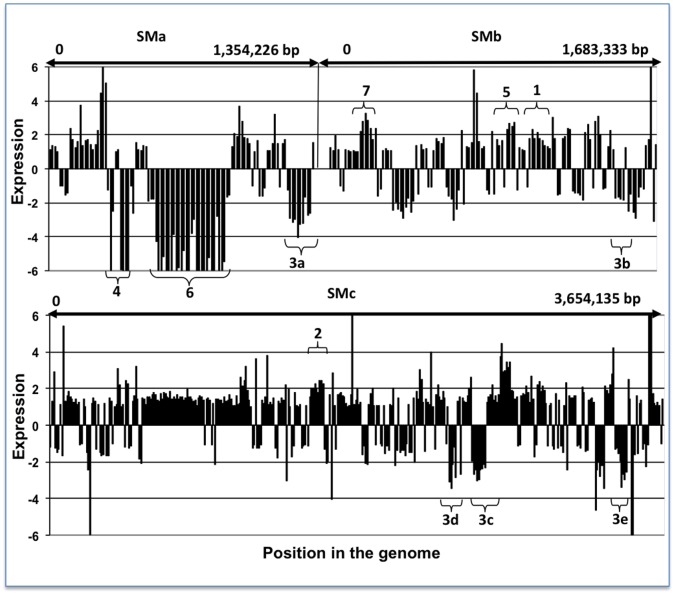
Expression profile of Rm1021 genes under low *vs.* high nitrogen. The expression ratio of the 609 genes identified in the study as either nitrogen stress responsive (Cluster I and II) and/or affected by mutations in the GlnD/GlnBK regulatory cascade (Cluster III). 1– *wgeA*, *wgdB*, *wgdA*, *wggR*, and *wgcA*; 2– *aap* operon; 3a – *rhs, rhb,* and *rhr* region; 3b – SMb21429, SMb21430, SMb21431, SMb21432– Putative iron ABC transporter; 3c – *hmu* region; 3d – SMc01657, SMc01658, SMc01659; 4– *nosR/Z*, *nnrU* – denitrification regulator protein; 5– *pho* region; 6– *fixN* region; 7– carbon fixation.

In a number of cases, we found linked sets of genes where some of the genes met the 2.0-fold change between conditions with a *P* value of <0.05 but others did not or where the genes met the criteria in one mutant but not the wild type. In the cases like those considered below, the expression trended in the same direction as adjacent genes but did not meet the numerical standards (Tables S2 and S3). We grouped all 609 genes based on their location as a part of probable or known operons or by related functions. Many of these groups of genes had the same expression pattern in the *glnBglnK* mutant background. For example, genes *wgeA*, *wgdB*, *wgdA*, *wggR*, and *wgcA*, which are involved in galactoglucan biosynthesis and secretion, belong to a single transcriptional unit [19]. Two genes, *wgdB* and *wggR*, met the 2X criterion for being described as upregulated in Rm1021 under low nitrogen (2.0-fold cutoff with a *P* value of <0.05) (Table S2, Column F, Rows 41,43). Three others, *wgeA*, *wgdA*, and *wgcA* also had increased expression under nitrogen limitation but these differences did not meet the initial criteria because their induction ratio under low nitrogen was between 1.75 and 2.0 (Table S2, Column F, Rows 40,42,44) and the *P* value of *wgeA* was 0.08 (Table S2, Column E, Row 40; Fig. 1. 1). All five genes were upregulated in Rm1021Δ*glnB*Δ*glnK* relative to Rm1021 under nitrogen excess (2.0-fold cutoff with a *P* value of <0.05) (Table S2, Column L, Rows 40–44; Fig. 1.1). Based on the linkage of these genes and their coordinate regulation, we assigned all five of these genes to a single group, and concluded that their expression in Rm1021 is upregulated under nitrogen limitation and repressed by PII proteins under sufficient nitrogen. A similar expression pattern was described previously for glutamine synthetase II (GSII) [9], [11].

Another example of a heterogeneous group was the *aap* operon where 3 out of 4 genes, *aapJ, aapQ, and aapM*, were upregulated under nitrogen limitation in Rm1021 (2.0-fold cutoff with a *P* value of <0.05) and one, *aapP*, had expression ratio 1.8 with P value <0.0001 (Table S2, Column F, Rows 26–29; Fig. 1.2). All four ORFs were also treated as a single group upregulated under nitrogen limitation in Rm1021, with the expression level repressed in the presence of PII proteins under sufficient nitrogen. Similar grouping was used to include as NSR genes those that just missed the quantitative criterion for NSR regulation when considered as isolated genes. This approach increased the total number of genes considered as differentially expressed in Rm1021 cells grown under nitrogen limitation to 88 that were upregulated and 56 that were downregulated.

Twenty-one genes related to iron uptake were differentially expressed in ammonium grown cells of Rm1021Δ*glnD-*sm2 *vs.* Rm1021Δ*glnB*Δ*glnK* (2.0-fold cutoff with a *P* value of <0.05) (Table S2, Column P). For example, the 14 kb pSymA *rhs, rhb,* and *rhr* region with genes involved in rhizobactin synthesis and regulation (Table S2 Rows 139–146,; Fig. 1.3a,), a 4 kb chromosomal region with genes involved in iron transport (*foxA*, *fhuFP*) (Table S2 157–159; Fig. 1.3d), a 4 kb pSymB region containing putative iron transport proteins (Table S2, Rows 148–151; Fig. 1.3b), and a 7 kb chromosomal region with genes encoding a heme compound transporter (*hmu*) (Table S2, Rows 152–156; Fig. 1.3c) were expressed at a significantly higher level in Rm1021Δ*glnD-*sm2 than in Rm1021Δ*glnB*Δ*glnK*. All of these genes had at least 2 fold decreased expression in Rm1021 cells grown on glutamate vs. cells grown on ammonium (*P* value <0.2). This *P* value was too high to consider these genes to be differentially expressed if they were considered to be single units. But when the genes were grouped together based on location (Fig. 1. 3a, 3b, 3c, and 3d) and treated as a single unit, the statistical analysis showed that these genes were differentially expressed with a *P* value of <0.05. A similar approach was applied to the groups of genes involved in nitrogen and phosphate metabolism (Fig. 1.4 and 1.5).

### Validation of Microarray Expression by qRT-PCR

**Figure 2:**
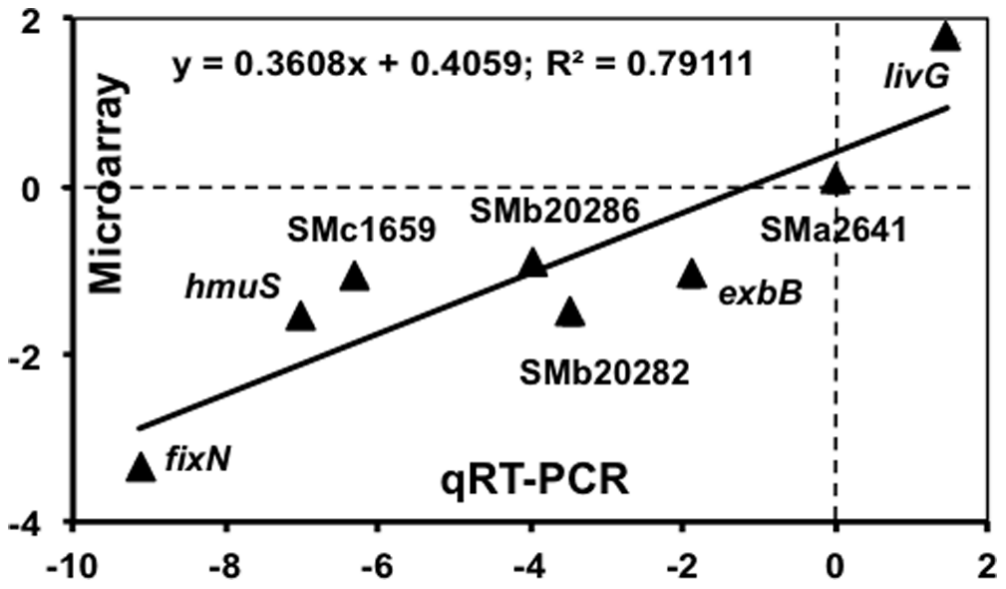
Comparison of log_2_-transformed microarray and qRT-PCR data of 7 representative genes differentially expressed in Rm1021 cells under high *vs.* low nitrogen.

qRT-PCR was used to confirm the microarray expression data. SMb20282, *livG*, and *fixN* were chosen based on the expression ratio in the cells grown on glutamate vs. ammonium. Two others belonging to the iron metabolism groups, *hmuS* (3c) and SMc01659 (3d), were considered as differentially expressed based on the cluster analysis described above. The regression of the microarray data and the PCR data indicated a good correlation between the data (R^2^ = 0.79) (Fig. 2).

**Figure 3:**
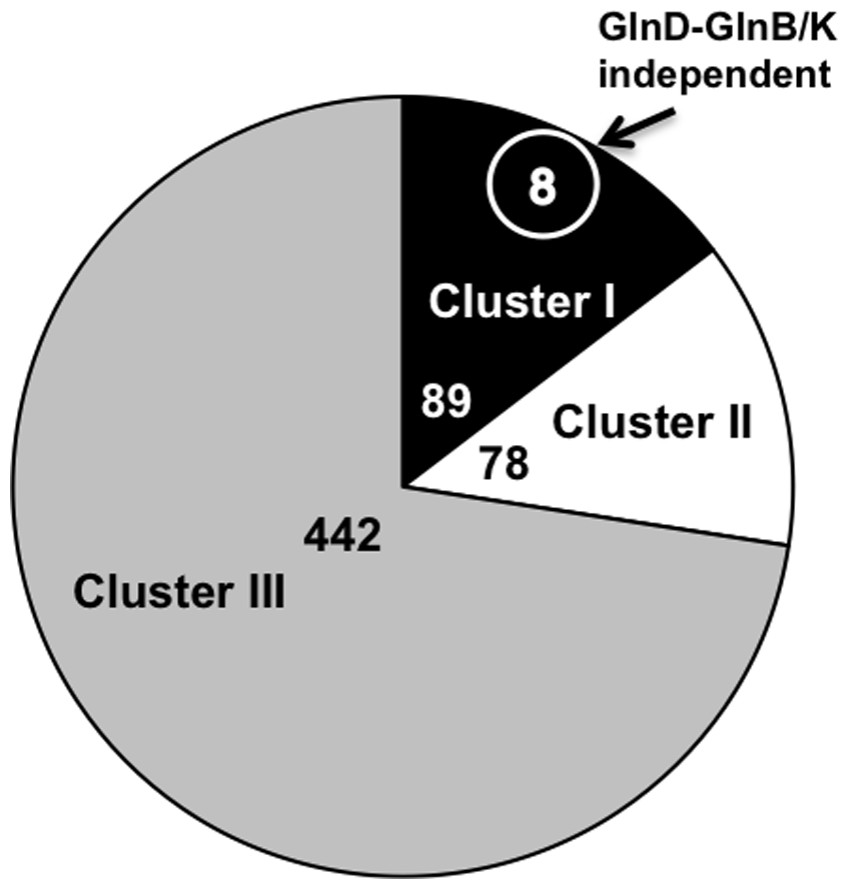
Distribution of differentially expressed genes in miroarray profiles. Cluster I, genes upregulated under nitrogen limitation; GlnD-GlnB/K dependent; Cluster I (circle), genes upregulated under nitrogen limitation, GlnD-GlnB/K independent; Cluster II, genes downregulated under nitrogen limitation; Cluster III, genes with expression affected by GlnD/GlnBK regulatory cascade but not differentially expressed under high *vs.* low nitrogen.

As a result, of the 609 genes identified as differentially expressed in one or more transcription profiling conditions, we concluded that expression of 165 was affected by nitrogen availability. These comprised <2.75% of the total number of ORFs annotated in the Rm1021 genome. 89 genes were upregulated under nitrogen limitation (Cluster I) and 78 were downregulated (Cluster II) (Fig. 3; Indicated in **Bold** in Table S2). These genes are part of a basal NSR in *S. meliloti*. The remaining 442 genes were not a part of the NSR *per se* but their regulation was significantly influenced by the mutations in GlnD or/and the PII proteins (Cluster III) (Fig. 3; Table S3).

### Distribution Analysis of Differentially Expressed Genes in *S. meliloti* Rm1021 Replicons

**Figure 4:**
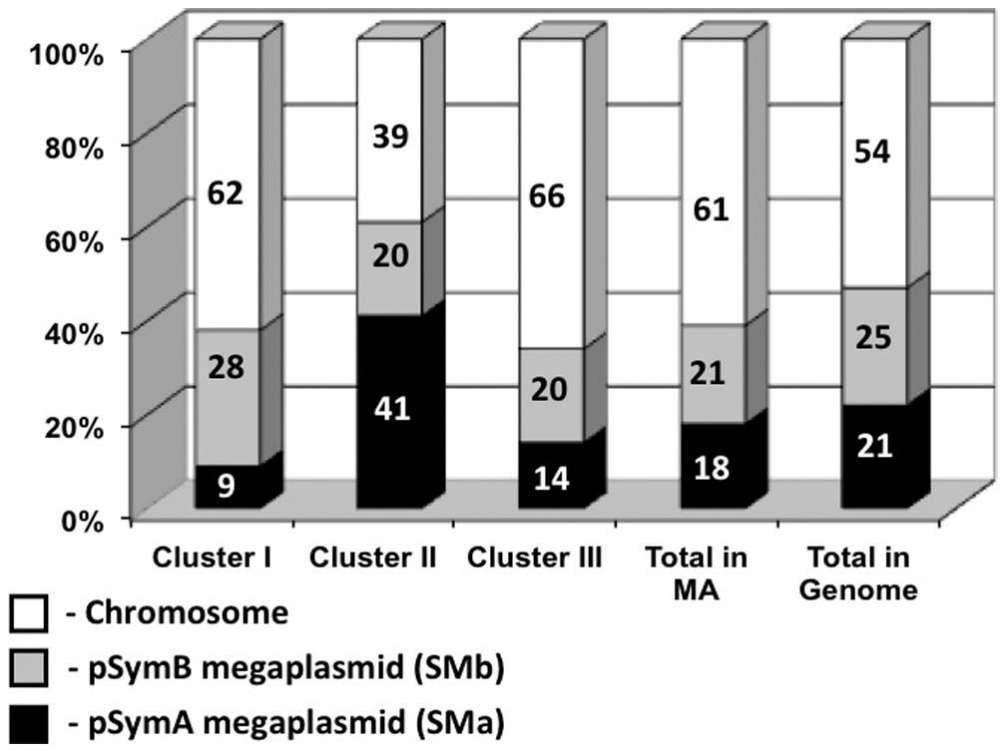
Distribution of differentially expressed genes in *S. meliloti* Rm1021 replicons. Cluster I. Genes upregulated under nitrogen limitation (97); Cluster II. Genes downregulated under nitrogen limitation (78); Cluster III. genes with expression affected by GlnD/GlnBK regulatory cascade but not differentially expressed under high *vs.* low nitrogen (442). Total in MA (microarray). The distribution of the 609 genes found to have differential regulation in response to nitrogen stress or mutations in *glnD* or *glnBglnK*, as described in the text. Total in Genome. Distribution of annotated genes on pSymA, pSymB or the chromosome.

Our analysis suggested a strong replicon bias in the distribution of the NSR genes (Fig. 4). The pSymA symbiotic megaplasmid had 10 upregulated genes (9% of all upregulated genes). This was 2.3-fold less than expected, since 21% of all predicted protein encoding genes are on pSymA [13]. On the other hand, 34 of the downregulated genes were located on pSymA, 44% of all downregulated genes and twice the expected proportion. While the ratio between upregulated and downregulated genes with chromosomal (SMc) localization was more than 2, the ratio between upregulated and downregulated genes located on pSymA was ∼0.26.

Additionally, our analysis revealed that a ∼120 kb region of pSymA (SMa1077–SMa1297) was affected by nitrogen availability (Fig. 1.6). 23 of the 123 genes in this region were repressed in the cells grown on glutamate as nitrogen source (2.0-fold cutoff with a *P* value <0.05). This was ∼25% of all genes downregulated under nitrogen limitation and ∼60% of downregulated genes on pSymA. This region includes a 53-kb segment rich in genes annotated as encoding proteins related to nitrogen metabolism, including an entire denitrification pathway and the *fix-1* cluster [20].

### Functional Classification of Differentially Expressed Genes

Based on genome annotation, a significant fraction of all differentially expressed genes identified in the study were genes annotated as transport proteins (29%) and proteins with unknown or hypothetical functions (30%) (Table 2). A very small number of differentially expressed genes that were downregulated under nitrogen stress were in the functional classifications related to small molecule metabolism, like amino acid metabolism and transport. On the other hand, most differentially expressed genes within the functional classification of nitrogen fixation and nodulation were downregulated. The majority of the genes within the functional classifications of nitrogen metabolism and transport were upregulated by low nitrogen (Table 2). Interestingly, the expression of the majority of differentially expressed genes annotated as being involved in shock adaptation (chaperonins) and mobility and chemotaxis were not affected by nitrogen availability, but were affected by the mutations in the GlnD or GlnB/K regulatory proteins (Cluster III).

**Table 2:** Functional classification of differentially expressed genes with >2.0 fold changes.

Functional classification	Total	Cluster 1	Cluster II	Cluster III
Small Molecule Metabolism^1^	86	17	2	66
Amino Acid metabolism	26	10	3	13
Nitrogen metabolism	9	6	1	3
Nitrogen fixation/nodulation	14	0	11	3
DNA/RNA metabolism/Chaperonins	15	0	0	15
Surface polysaccharides/cell envelope	17	7	1	9
Mobility chemotaxis	32	0	0	32
Transport of small molecules^2^	63	7	2	54
Transport of nitrogen containing compounds	88	27	7	54
Transport of iron containing compounds	22	0	19	3
Not classified regulator	18	2	3	13
Hypothetical/Global homology	163	10	23	130
Others	56	3	6	47
Total	609	89	78	442
Phosphate stress response^3^	156	25	25	106
Putative Pho boxes^4^	20	2	1	17

1– Functional categories “Amino Acid metabolism” and “Nitrogen metabolism” are not included; 2– Functional categories “Transport of nitrogen containing compounds” and “Transport of iron containing compounds” are not included; 3– as identified by Krol and Becker [57]; 4– genes with promoter region containing Pho-box identified by Yuan et al., [58].

### Global Nitrogen Stress Response (Table S2)

#### Cluster I comprised the genes upregulated under nitrogen limitation (Table 2)

Carbon metabolism. Only 8 of the 165 genes whose expression in Rm1021 was affected by nitrogen availability had expression that was not substantially affected by the mutations in *glnD* or *glnBglnK* [Cluster I.IV (Table S2, Fig. 1.7)]. These include *S. meliloti* genes that are part of the reductive pentose phosphate pathway or Calvin Benson Bassham cycle (CBB cycle): ribulose-phosphate 3-epimerase SMb20195, *ppe* ATPase, *cbbSL* small and large subunits of the RuBisCO complex, *cbbA* fructose-1,6-bisphosphate aldolase, *cbbT* transketolase, and *cbbP* phosphoribulokinase. This pathway is the most important mode of autotrophic CO_2_ fixation in nature. Formate-dependent autotrophic growth of Rm1021 relies on the presence of the *cbb* operon, as well as formate dehydrogenase (*fdsABCDG)* and a triose-phosphate isomerase, *tpiA* or *tpiB* [21]. This indicates that expression of the *cbb* operon was unique in being induced under nitrogen limitation but in a way that was not directly coupled to the NSR regulated by GlnD or the PII proteins. We also found several putative sugar transporters and enzymes involved in carbon metabolism whose expression was affected by GlnD or/and GlnB/K but was not affected by nitrogen limitation (Table S3).

Previously it was reported that the presence of glutamate induced a rearrangement of gene expression involved in carbon metabolism [22]. Our expression analysis did not completely overlap with the data from the previous report, probably because of the difference in carbon sources used for cell growth. However, sugar transporters SMb20902, SMb20903, SMc02514 and a lipoic acid related gene, *lpdA2* (2-oxoglutarate dehydrogenase E3 component), were differentially expressed in both studies.

Glutamine synthetase II (glnII) was upregulated under nitrogen limitation, which was consistent with the previously published data [1], [9], [22], [23] and with measures of the presence of the GSII enzyme. Using a translational fusion to the *glnII* promoter we had previously found that Rm1021Δ*glnB* had a high level of GSII expression regardless of the availability of nitrogen in the media, which indicated that deletion of *glnB* resulted in constitutive GSII production [9]. Our microarray data are in agreement with this conclusion – the expression of GSII was upregulated in the Rm1021Δ*glnB*Δ*glnK* mutant grown on ammonium.

The microarrays indicate that a small protein, located upstream of *glnII* and annotated as the *gstI* glutamine synthetase translation inhibitor, was repressed in Rm1021 grown on glutamate (Cluster II) (3 fold P value 0.07). This was consistent with the proposed function of the protein [24]. The expression of *gstI* was also repressed in Rm1021Δ*glnB*Δ*glnK,* and would be predicted to allow a high level of GSII production, as we reported earlier [9].

**Figure 5:**
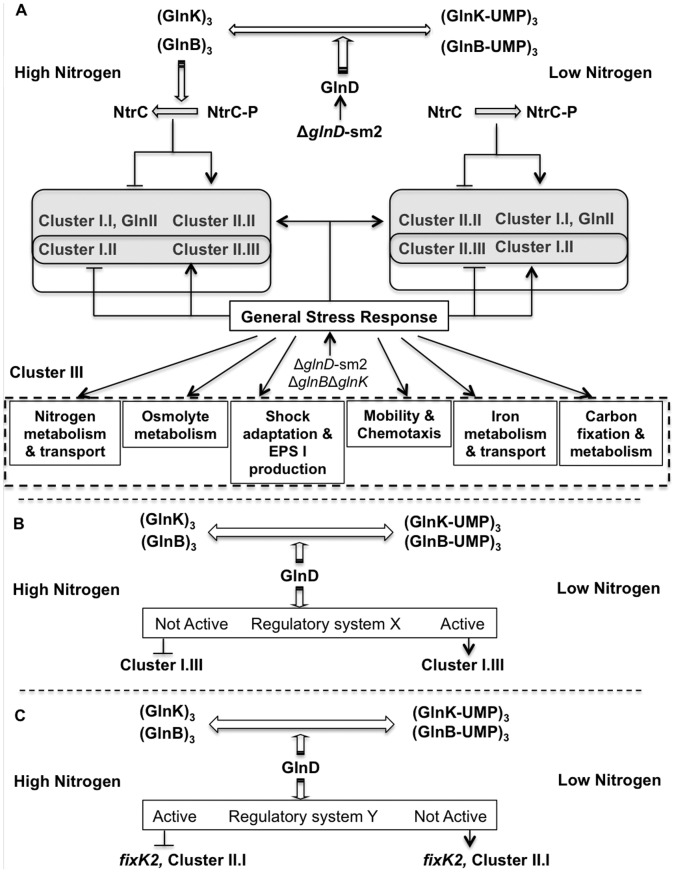
Model of GlnD and GlnBK regulation in *S. meliloti* Rm1021. A – GlnD acts strictly through PII proteins to regulate gene expression. General Stress Response affects the expression of several clusters of these genes. B, C – GlnD acts through the PII proteins as well as through PII independent regulation to alter gene expression.

A large group of ORFs had an expression pattern similar to *glnII* (Table 2; Cluster I.I); they were upregulated under nitrogen limitation in wild type cells and were constitutively expressed in the *glnBglnK* mutant (Fig. 5. A). This cluster includes a number of genes involved in metabolism of nitrogen containing compounds and includes the *ntrC/ntrB* two component regulatory system, the *amtB* ammonium transporter, nitrate transporters SMa0581-0583-0585 and SMb21114, small and large subunits of nitrate reductase SMb20984-20985, genes involved in biosynthesis of cysteine (*cysN/D/H*) and methionine (*metH*), and the transport of histidine (*hisX*), arginine (SMc03125), and proline-betaine (SMc01642/3/4). This group also contained putative amino acid transporters (SMc04037, SMc02356), a general L-amino acid transport system (*aapJQMP*) specific to amino acids with a polar side chain [25], and a putative dipeptide transporter (*dppA_2_B_2_*). An important methyl donor for transmethylation and polyamine biosynthesis adenosylmethionine synthetase (*metK*), was also found in this group. Additionally, Cluster I.I contained several genes involved in galactoglucan biosynthesis and secretion, *wgeA*, *wgdB*, *wgdA*, *wggR*, and *wgcA* [19], [26] and several ABC transporters and hypothetical proteins.

The simplest model that can explain the expression pattern of Cluster I.I is in agreement with the model we proposed earlier [12], which hypothesizes that (i) under high nitrogen, unmodified PII proteins are required to stimulate NtrC-P dephosphorylation resulting in downregulation of the genes; (ii) in the absence of PII proteins NtrC is always phosphorylated, resulting in the constitutive expression of these genes (Fig. 5. A). The *glnD* mutation decreased the effect of nitrogen availability on the expression of most of these genes.

Surprisingly, the expression of *glnII* was not significantly affected by the *glnD* deletion – it was still upregulated under nitrogen limitation. Additionally, the expression of some other genes, including the *nirD* nitrite reductase, SMb21114 putative nitrate transporter, and the *amtB* ammonium transporter, was also affected by nitrogen limitation in Rm1021Δ*glnD-*sm2 suggesting the existence of another regulatory mechanism of NSR. We showed previously that Rm1021Δ*glnD-*sm2 does not produce a detectable level of GSII in the cells grown under either high or low nitrogen [11]. The decreased production of GSII in the *glnD* background could not be explained by an overexpression of *gstI* on glutamate – this gene was still downregulated in Rm1021Δ*glnD-*sm2 under nitrogen limitation (2.6 fold with P value 0.1). One possible explanation could be that in the *glnD* background GSII undergoes posttranslational modification resulting in its degradation.

It was reported that a single *glnB* deletion downregulated GSII expression in glutamate grown cells [22]. In our microarray analysis, we did not detect a similar effect of the *glnBglnK* deletion on the expression of GSII and the majority of the genes listed above. However, in agreement with the earlier report [22], the *amtB* ammonium transporter, as well as *metH* and *metK*, were downregulated in Rm1021Δ*glnB*Δ*glnK* in cells grown on glutamate.

Cluster I.II. The high-affinity branched-chain amino acid transport system (*livKFGMH*) had an expression pattern distinct from GSII. It was highly induced in the *glnBglnK* mutant grown on ammonium and it was strongly repressed in the Rm1021Δ*glnD*-sm2 mutant in both high and low nitrogen. However, this repression did not abolish the differential expression of the system – the expression of *livKFGMH* in the *glnD* mutant was still 2.5–4 times higher under nitrogen limitation. The differential expression of *livKFGMH* genes might be a result of the activation of a **g**eneral **s**tress **r**esponse (GSR) in the *glnD* mutant. Previously we showed that Rm1021Δ*glnD*-sm2 had more severe growth defects when grown on glutamate as nitrogen source compared to growth on ammonium [11], suggesting higher activation of GSR in cells grown on glutamate. Consequently, the increased expression of *livKFGMH* on glutamate in the *glnD* mutant might be a part the GSR (Fig. 5. A).

LivKFGMH proteins are highly homologous to the BraDEFGC broad-specificity amino-acid transporter involved in the transport of branched-chain amino acids in *R. leguminosarum* [27]. Together with AapJQMP, LivKFGMH was shown to be involved in cycling amino acids between the plant host and mature bacteroids in the *R. leguminosarum*-pea symbiosis – a process critical for the ability of these rhizobia to establish effective symbiosis [28], [29]. In agreement with our data, *liv* and *aap* genes were also shown to be upregulated in nitrogen starved *S. meliloti* cells by Dovalos et al. [22]. Based on our microarray data the expression of the *aap* operon is strictly regulated by the GlnD/GlnBK regulatory cascade (Cluster I.I). However, the regulation of expression of the *liv* operon is more complicated and it appears to involve another, and unknown, regulatory component, in addition to GlnD/GlnBK.

Cluster I.III. is comprised of genes with expression downregulated in Rm1021Δ*glnB*Δ*glnK* and Rm1021Δ*glnD-*sm2 strains under low nitrogen. This group included several genes involved in methionine transport and biosynthesis, such as *metQ*/*metI* transporters, betaine-homocysteine methyltransferase (*bmt*), and probable methylenetetrahydrofolate reductase oxidoreductase (*metF*). Additionally, this cluster included probable adenosylhomocysteinase (SMc02755), putative phosphoglycerate dehydrogenase (*serA*), and succinoglycan regulator *exsB* [30], [31]. The expression pattern of Cluster I.III suggested that (i) there is another regulatory system that represses these genes in the absence of PII proteins; and (ii) the expression of this system is controlled by GlnD in a GlnB/K independent manner (Fig. 5. B).

#### Cluster II is comprised of genes upregulated under high nitrogen (Table 2)

Some of these genes were downregulated under nitrogen limitation in the *glnBglnK* and *glnD* mutants (Cluster II.I). Several genes essential for symbiotic nitrogen fixation were found in this group, including transcriptional regulators *fixT_1_*, *fixT_2_*, *fixK* and a *fixK-*like regulator (SMa1207), the nitrogen fixation protein, *fixH*, an iron sulfur membrane protein, *fixG*, and a *cbb_3_*−type cytochrome oxidase complex, *fixN_1_O_1_Q_1_*.

The expression of *fixK*, *nifA*, *proB* and SMc03253 is directly regulated by FixJ – a global regulator controlling the expression of nitrogen-fixation genes in response to a low oxygen environment [32], [33]. In our microarray, we detected a very high level of *fixK_2_* induction in wild-type cells grown on ammonium (>20 fold). Additionally, L-proline cis-4-hydroxylase (SMc03253) and putative glutamate 5-kinase (*proB2*) were also upregulated in Rm1021 on ammonium (>19 and >10 fold respectively). *nifA* had >2.5 fold induction under high nitrogen in Rm1021 but because of the high *P* value (∼0.08) it was not considered to be differentially expressed in the wild type strain (Cluster III).

It was very surprising to find that expression of some of the direct targets of the FixJ protein were affected by nitrogen availability in addition to the previously reported regulation by oxygen. However, there is evidence showing that in *S. meliloti* reactive nitrogen intermediates (RNIs) can serve as signaling molecules, and there is an overlap/interaction between NO-mediated control and FixLJK-mediated microaerobic signal transduction [34]. In our microarray we found that the *nrtABC* nitrate transport system and nitrite reductase *nirBD* are upregulated under nitrogen limitation, which could lead to an increased NO concentration in glutamate grown cells.

In *S. meliloti*, *fixK* controls expression of 97 genes involved in the free-living and symbiotic life-styles [35], including *fixN_1_O_1_Q_1_P_1_*, *fixO_2_Q_2_P_2_*, *fixGHIS*, *fixT_(1,2)_*, SMa1207, *fixM*, *hspC2*, SMa1147, SMa1158, and SMa1231, which were found in Cluster II.I. In *Bradyrhizobium japonicum fixK2* is a critical regulator in the FixLJ-dependent regulatory cascade. In addition to controlling the expression of *fix* genes, *rpoN*, nitrate respiration and heme biosynthesis genes, *fixK_2_* is responsible for activating *fixK_1_* [36]. The axis of symmetry of a classical FixK box (5′-TTGANNNNNNTCAA-3′) was found between bases −41 and −40 relative to the transcription start of fixK1 [36], [37]. It is not clear whether FixK_2_ activates *fixK_1_* in *S. meliloti* and no obvious FixK boxes are found in the *fixK_1_* promoter [35]. We did not detect upregulation of *fixK_1_* under high nitrogen.

In total, 39 putative FixK boxes were found in *S. meliloti* pSymA, 16 of the genes with promoters containing a FixK box were found near the 5′ end of Cluster II.I genes, and 5 were near the 5′ end of Cluster III genes (Table S4). This suggested that FixK might be involved in regulating the expression of some of the genes comprising Cluster II.I.

The D,L-2-aminoadipic acid transport system (SMc03131, SMc03133, and SMc03135) was also induced in wild type cells and repressed in the *glnD* mutant under high nitrogen. D,L-2-aminoadipic acid acts as a glutamine synthetase inhibitor [38]. The induction of the transport system resulting in increased uptake of the compound under nitrogen excess might be another mechanism that decreases GS activity. The expression pattern of Cluster II.I genes mirrored the expression pattern of Cluster I.III genes and can be explained by a similar model (Fig. 5. C).

Cluster II.II is comprised of 8 genes located together (SMb20278-SMb20285) that, in addition to being repressed under nitrogen limitation in the *glnBglnK* mutant, were also upregulated in the *glnD* mutant under both high and low nitrogen. This indicated that the mutation in GlnD protein caused an altered expression of an unknown regulator that stimulates expression of this cluster (Fig. 5. A). This group is comprised of a *lysR* regulator, a putative polyamine (spermidine/putrescine) ABC uptake system homologous to *potFHIG* [39], a pyridine nucleotide-disulphide oxidoreductase (SMb20280), a hypothetical protein (SMb20279), and carboxymuconolactone decarboxylase (SMb20278). Polyamines exist mostly as polyamine-RNA complexes in cells and are therefore presumed to play a critical role in regulating cellular functions including protein translation and cell cycle progression [40]. It was shown that, along with other effects, polyamines affect the NSR by enhancing the synthesis of RpoN (σ^54^), stimulating flagellin synthesis, oligopeptide uptake (OppA), and iron transport at the level of translation in *E. coli* cells grown on glutamate [40]–[42]. A number of the genes involved in these functions were found in our microarray analysis (see below). While the expression of putative spermidine/putrescine transporters (*potFGH* and Smc01965-SMc01966), putative putrescine and agmatine ABC transporter (SMc01652, SMc01654), and *speB2* putative agmatinase involved in polyamine biosynthesis were not affected by nitrogen stress, they were upregulated in the *glnBglnK* mutant under high nitrogen (Cluster III). These data indicate that regulation of the polyamine pool is a complex process that relies on the function of NSR, and altered polyamine metabolism might be another tool allowing *S. meliloti* to adapt to changing nitrogen availability.

Cluster II.III is comprised of 22 genes involved in iron metabolism, including siderophore biosynthesis (*rhbABCDEF*, *rhtA*, *rhrA*, and SMa2339), iron uptake system (*fbpA* and SMb21429-21430-21431-21432), hemin transport system (*hmuPVUTS* and *hmuR*), Ton-dependent siderophore receptor (*fhuA2*), and ferric-iron reductase SMc01658. This cluster also included a putative translational regulator SMc02888 and a putative ABC transporter SMc01659. The deletion of PII proteins significantly decreased expression of these genes in ammonium-grown cells and shifted the expression ratio towards upregulation under low nitrogen. On the other hand, the mutation in *glnD* had no effect on the ratios of expression of these genes – the mutated strain had even higher ratio of gene expression in ammonium grown cells vs. glutamate grown cells. This is another group of genes with expression that is probably controlled by both the GlnD-GlnBK regulatory cascade, and another regulatory system that is a part of GSR (Fig. 5. A).

### Cluster III is Comprised of 442 Genes whose Expression was not Affected by Nitrogen Limitation but was Affected by Mutations in GlnD or the PII Proteins (Table S3)

The differential expression of these genes may not be directly linked to the function of the GlnD or PII proteins, but could result from nutritional limitations that result in the decreased growth rate of the mutant strains. Surprisingly, the *glnD* gene itself was found in Cluster III. The expression of *glnD* was downregulated in Rm1021Δ*glnD*-sm2 both in high and low nitrogen but its expression was not affected in Rm1021Δ*glnB*Δ*glnK*. This expression pattern most probably is not a result of nutritional limitation but instead indicates that GlnD plays role in regulating its own expression in a GlnB/GlnK-independent manner. Several functional groups were found in Cluster III, such as (i) metabolism and transport of nitrogen containing compounds, (ii) osmolyte metabolism, (iii) shock adaptation, (iv) mobility and chemotaxis, (v) carbon metabolism, and (vi) succinoglycan (EPS I) production (Table S3, Fig. 5. A).

A number of genes involved in amino acid biosynthesis were found in Cluster III, including glutamate synthases (*gltB* and SMc01814), putative glutamine synthetase type I (SMc01594), glutamine synthetase III (*glnT*) and an aspartate aminotransferase (SMc02251). The expression of several amino acid and oligopeptide transporters (*tauABC*, *oopABCD,* SMc02357–2359, SMc02257-02258-02260, SMc00138–00139, *hypNMPQ*) was affected by mutations in the GlnD-GlnBK regulatory cascade. The transporters for heme precursor delta-aminolevulinic acid, *dppABCF* [43] and its second copy *dppC_2_D_2_F_2_*, and for a periplasmic dipeptide transport protein (SMc02025) were upregulated in the ammonium grown *glnBglnK* mutant. Additionally, the *glnD*-*mviN*-SMc01122 operon, *nifA* transcriptional activator, *nozR* regulator, *nosZ* N_2_O reductase, and *nnrU* denitrification regulator were differentially expressed in the GlnBK or GlnD mutants.

Interestingly, several genes involved in osmolyte metabolism were also found in Cluster III including a putative glycine-betaine and choline ABC transporter (SMc02344), ABC-type glycine-betaine transporters (SMa1462, SMb21572), homologues to *prbB* proline-betaine uptake system (SMa1862), *choW* choline transporter, trehalose/maltose transport system (*thuEFGK*) and trehalose catabolism protein (*thuA*). The altered expression of these genes might be a part of adaptation to osmotic stress caused by misbalanced nitrogen metabolism in *glnD* and *glnBglnK* mutants. However, it also could be a part of altered carbon and nitrogen metabolism.

As a part of GSR caused by GlnD-GlnB/K mutations, a number of chaperonins, heat and cold shock proteins, including *groEL2*, *groEL5*, *htpG*, *hslU/V*, *ibpA*, and *clpB*, and genes involved in detoxification, including major H_2_O_2_-inducible catalase (HPII) *katA* [44], and *metF_1_/F_2_/E_2_* probable multidrug-efflux system were found in Cluster III.

Thirty-one genes involved in motility and chemotaxis were downregulated in Rm1021Δ*glnB*Δ*glnK* and Rm1021Δ*glnD-*sm2 under nitrogen limitation, which is in agreement with previous data on the repression of motility and chemotaxis genes by a variety of environmental stresses and nutritional starvation [45]–[47].

### Succinoglycan (EPS I) Production

Nitrogen availability has been found to significantly affect biosynthesis of succinoglycan (EPS I) in Rm1021 [48], [49]. The SyrM protein directs this, acting as positive regulator under nitrogen starvation [50], [51]. *exsB* gene encoding a succinoglycan biosynthesis regulator [30], [31], was upregulated in glutamate grown Rm1021 (Cluster I.III). ExsB is a negative regulator, and a mutation in *exsB* caused a 3 fold increase in EPS I production. However, an additional copy of *exsB* had a minor effect on EPS I production and no effect on *exo* gene expression. Negative regulation of EPS I production by ExsB has been suggested to occur post-translationally [52].

**Figure 6:**
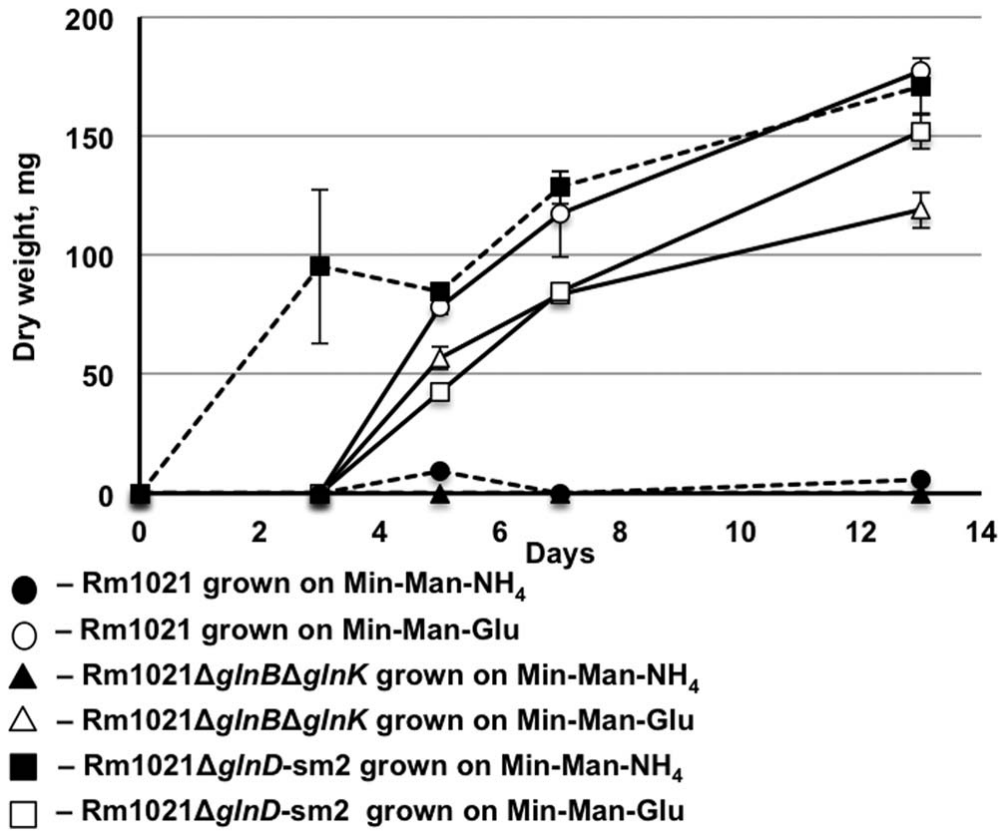
HMW EPS I production by *S. meliloti* strains. Strains were grown in MM media with glutamate or NH_4_ as nitrogen source. The EPS I were harvested after 3, 5, 7 and 13 days and dried.

Surprisingly, several genes involved in EPS I biosynthesis (*exoA*, *exoO*, *exoN, exoF_1_*, and *exoY*) were upregulated in Rm1021Δ*glnD-*sm2 grown under high nitrogen, compared to their expression level in Rm1021 and Rm1021Δ*glnB*Δ*glnK*. The key succinoglycan biosynthesis gene, *exoY*, appears to be the primary target of regulation of EPS I production [53]–[56]. To test whether *glnD* deletion affected EPS I production we evaluated the ability of Rm1021, Rm1021Δ*glnD-*sm2 and Rm1021Δ*glnB*Δ*glnK* to produce high molecular weight (HMW) EPS I under high and low nitrogen availability. We detected a high level of HMW EPS I production by all three strains grown under nitrogen limitation (Fig. 6). EPS I production was shut down when Rm1021 and Rm1021Δ*glnB*Δ*glnK* strains were grown in the presence of ammonium. On the other hand, ammonium-grown Rm1021Δ*glnD-*sm2 produced EPS I at a level similar to glutamate-grown Rm1021Δ*glnD-*sm2 (Fig. 6).

We have shown that the truncated GlnD in Rm1021Δ*glnD-*sm2 is unable to modify PII proteins in nitrogen-limited cells [9] and interpreted this data as indicating that cells of Rm1021Δ*glnD-*sm2 were locked in the regulatory state characteristic of high nitrogen status, regardless of actual nitrogen availability. The overexpression of *exo* genes and overproduction of EPS I in ammonium-grown Rm1021Δ*glnD-*sm2 indicate that regulation of EPS I production is not strictly controlled by the GlnD/GlnBK regulatory cascade, but involves other regulatory mechanisms which are activated in nitrogen starved Rm1021Δ*glnD-*sm2.

### Co-regulation of Nitrogen and Phosphate Stress Response

**Figure 7:**
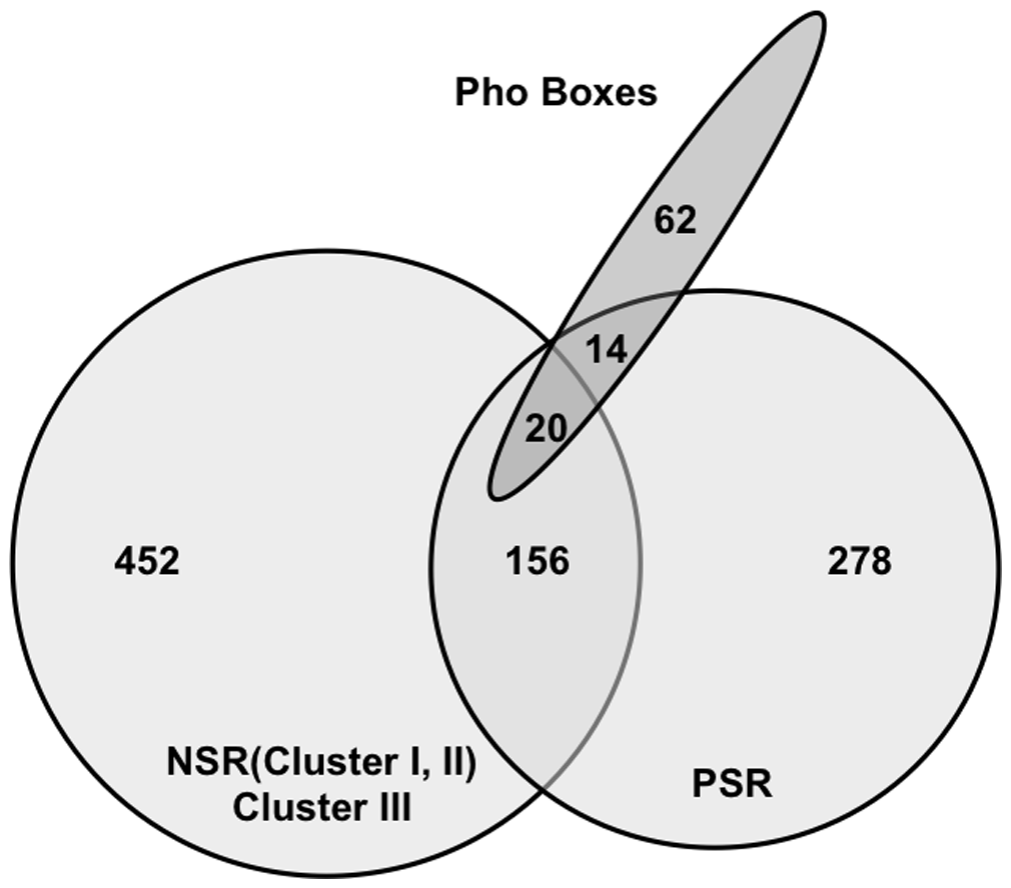
Overlap between nitrogen and phosphate stress responses. Left circle –609 genes were identified in our study that were regulated in response to nitrogen stress or had significantly different expression in Rm1021Δ*glnD*-sm2 or Rm1021Δ*glnB*Δ*glnK*. Right circle –435 genes found to be differentially expressed under phosphate starvation in Rm1021 or/and Rm2011 [57]; Overlap –156 genes were found to be regulated by the conditions in both studies. Ellipse –96 putative Pho regulon members were identified [58], 34 Pho regulon members were differentially expressed under phosphate starvation [57]. Of these 34 genes, 20 were identified as differentially expressed in our microarrays by both sets of conditions.

An analysis of the global phosphate stress response in *S. meliloti* identified 435 genes that were differentially expressed in Rm1021 or/and Rm2011 under phosphate starvation [57]. 156 genes identified in our microarray analysis were part of the global phosphate stress response (PSR) (Table S5, Fig. 7). This was >25% of all differentially expressed genes identified in our microarray study and ∼36% of the global phosphate starvation response, which is a substantial overlap. Of these 156 genes, Krol and Becker [57] have previously shown that 38 had phosphate stress induced PhoB-dependent regulation, 20 were induced by phosphate stress in a PhoB-independent or partially PhoB-dependent way and 51 were repressed by phosphate stress independently of PhoB. 38 were not assigned to any clusters.

Pho regulon members in NSR. Recently, 96 putative Pho regulon members were identified whose promoter regions contained one or more Pho boxes, conserved motifs that bind the PhoB phosphate stress response transcriptional regulator [58]. 34 were differentially expressed under phosphate starvation (Table S6). Of these 34 genes, 20 (∼58%) were identified as differentially expressed in our microarrays (Fig. 7). Pho regulon members, including monomeric alkaline phosphatase (*phoX*) and the transcriptional activator of exopolysaccharide II (EPS II) synthesis (*wggR*, formerly *expG*), were induced under nitrogen limitation. Rhizobactin siderophore biosynthesis protein (*rhbF*) was repressed under nitrogen limitation and 17 other genes were differentially expressed in the *glnD* or *glnB/K* mutants.

Alkaline phosphatase PhoX and two putative alkaline phosphatases (SMc03242 and SMc01907) are strongly induced in a PhoB-dependent manner in *S. meliloti* grown under phosphate limitation [57]–[59]. In our microarray we found that *phoX* was also induced by nitrogen limitation and was strongly upregulated under high nitrogen in the strain missing the PII proteins. The expression of SMc01907 was not affected by nitrogen limitation in Rm1021 but it was upregulated under high nitrogen in the *glnBglnK* mutant.

An expression pattern similar to *phoX* was observed for EPS II transcriptional activator *wggR*, which is involved in the PhoB-dependent activation of the genes encoding EPS II biosynthetic proteins [19], [45], [60]. EPS II biosynthetic genes *wgeA*, *wgdB*, *wgdA*, and *wgcA* were also upregulated in Rm1021 cells grown on glutamate and in Rm1021Δ*glnB*Δ*glnK* grown on ammonium.

A number of Pho regulon gene family members involved in phosphate uptake and metabolism, such as the phosphate uptake systems *phoCDET* and *pstS-pstC*, phosphonate metabolism *phnNM*, phospholipase C SMc00171, *btaA-btaB* betaine lipid biosynthesis genes, phosphate binding periplasmic protein SMc02146, and putative polyphosphate kinase *ppk* were induced in Rm1021Δ*glnB*Δ*glnK* grown on ammonium (Table S5). Upregulation of genes that showed *phoB* dependent regulation induced by phosphate stress [57] in ammonium grown Rm1021Δ*glnB*Δ*glnK* might result from PSR activation caused by unbalanced nitrogen metabolism in the strain missing PII proteins. However, the possibility of cross talk between phosphate and nitrogen stress regulatory cascades should be considered.

PhoB independent PSR and NSR. Previously it was shown that a number of genes involved in iron uptake and metabolism were repressed in phosphate stressed *S. meliloti* independent of the *phoB* allele, which indicates phosphate independent stress related regulation involving another stress response regulator [57]. In our microarray experiments we found that iron uptake and metabolism were repressed in nitrogen stressed Rm1021 in a GlnD/GlnBK dependent manner (Cluster II.III). Additionally, the expression of a number of the genes involved in nitrogen metabolism and found to be controlled by the GlnD/GlnBK regulatory cascade (GSII, *aap* general L-amino acid and *liv* high-affinity branched-chain amino acid transporter), was also affected by phosphate limitation [57] (Table S5). This indicates that some branches of NSR might be activated under phosphate starvation.

### Conclusion

Our transcriptome analysis showed that the GlnD/GlnBK regulatory cascade in *S. meliloti* controls a larger group of genes and involves a wider range of processes than those that respond to nitrogen availability. This conclusion depends to some extent on the definition of nitrogen stress responsive genes as the observation of a two fold change between expression levels when cells are grown with ammonium or glutamate as nitrogen sources. But the much larger number of genes that are influenced by a mutated GlnD or deletion of the PII proteins strongly suggests that these regulatory proteins are involved in more than the nitrogen stress response. There are historical antecedents for this – enteric genes like *ntrBC* and *ropN/ntrA* that were originally identified through their participation in glutamine metabolism were later shown to have broader functions. The *S. meliloti glnD* gene appears to be essential, which is not the case in the enteric bacteria, an observation consistent with a larger role for GlnD in cell metabolism [61]. But an analysis of the influence of these genes on transcription in enterics does not appear to have been done.

Expression of genes, such as those involved in iron metabolism and transport, exopolysaccharide production, and *fixK* regulon expression, are affected by nitrogen limitation in a GlnD/GlnBK dependent manner. Additionally, our data suggested the existence of additional regulatory mechanisms that can participate in the *S. meliloti* NSR either through direct interaction with GlnD/GlnBK/NtrCB proteins or indirect regulation through starvation-related changes. Moreover, the transcriptome profile revealed a significant overlap between regulation in response to nitrogen and phosphate stresses. This overlap might be attributed to activation of a general stress response by alteration in nitrogen metabolism. However, the possibility of direct interaction between the GlnD/GlnBK/NtrCB regulatory cascade and the PhoR/PhoB two-component regulatory system should be considered.

### GlnD Protein: GlnB/GlnK Independent Mechanism of Action

Until recently, it was believed that, similar to enteric bacteria, the control of NSR in *S. meliloti* was strictly governed by GlnD through the GlnBK regulatory cascade by modification of the PII proteins, GlnB and GlnK [3]. Our previous studies showed that, in *S. meliloti,* GlnD can communicate with the cell by in ways that do not depend on the PII proteins [9], [11], [12]. The transcriptome analysis presented here is consistent with this conclusion.

Earlier we showed that GlnD-sm2, which is missing the N-terminal domain, is unable to modify PII proteins under nitrogen limitation [9]. If the only role of GlnD was to modify PII proteins in response to nitrogen availability, the transcriptome profile of Rm1021Δ*glnD*-sm2 should be similar to that of Rm1021 under high nitrogen conditions. Consequently, the genes that are a part of NSR (Cluster I and II) and are differentially expressed in ammonium grown Rm1021Δ*glnD*-sm2 and Rm1021 could be potential targets of GlnD-dependent regulation that does not depend on GlnB/K.

We have identified a number of potential targets for such regulation. Under high nitrogen, the expression of the *liv* high-affinity branched-chain amino acid transporter operon was repressed by GlnD truncation (Cluster I.II). Previous studies in *R. leguminosarum* showed that branched-chain amino acid transport was important in the regulation of nitrogen exchange the *R. leguminosarum*–pea symbiosis [62] and that mutational disruption of this transport contributed to a Fix+ Eff phenotype somewhat similar to the phenotype of Rm1021Δ*glnD*-sm2. Several genes essential for symbiotic nitrogen fixation, like the transcriptional regulators *fixT_1_*, *fixT_2_*, *fixK_2_* and *fixK-*like regulator (SMa1207), nitrogen fixation protein *fixH*, iron sulfur membrane protein *fixG*, and *cbb_3_*−type cytochrome oxidase complexes *fixN_1_O_1_Q_1_* (Cluster II.I) also have expression patterns that differ between Rm1021 grown in high nitrogen and Rm1021Δ*glnD*-sm2. In addition, genes thought to be involved in transport and metabolism of nitrogen containing compounds such as proline (*proB2*, L-proline cis-4-hydroxylase SMc03252), D,L-2-aminoadipic acid transport (SMc03131/03133/03135, Cluster II.I) and spermidine/putrescine transport (SMb20278–20285, Cluster II.II) might also be regulated by GlnD independently of GlnB/K. How this type of GlnD regulation works is unknown but it is possible that this proposed alternative to the classical regulation by GlnD through modification of the PII proteins is important in the reported essential function of GlnD in *S. meliloti*, a property not seen in the enteric bacteria.

## Supporting Information

Table S1 Gene-specific primers used for qPCR.
(DOCX)

Table S2 Genes that are regulated by nitrogen stress in *S. meliloti*.
(XLSX)

Table S3 Genes that are not regulated by nitrogen stress but where the expression is significantly influenced by mutations in GlnD or/and the PII proteins.
(XLSX)

Table S4 *S. meliloti* genes with promoters containing a FixK box that are regulated by nitrogen stress. *FixK Box as identified by Bobik *et al.* [35]; **Clusters as defined in this paper.
(XLSX)

Table S5 Genes that are regulated by both nitrogen and phosphate availability. *Clusters as defined in this paper; **Changes in gene expression under phosphate limitation (log2 ratios) [57]; ***Cluster I - Genes that showed phoB- dependent regulation induced by Pi stress; Cluster II - genes that showed phoB-independent or partially phoB-dependent regulation induced by Pi stress; Cluster III - genes that showed phoB-independent regulation repressed by phosphate stress; ****Pho Box as identified by Krol and Becker [57] and Yuan *et al.* [58].
(XLSX)

Table S6 Genes that are differentially expressed under phosphate starvation. *Changes in gene expression under phosphate limitation (log2 ratios) [57]; **Pho Box as identified by Krol and Becker [57] and Yuan *et al.* [58].
(XLSX)
