# Supplementary material for: Transcriptome Analysis of the Role of GlnD/GlnBK in Nitrogen Stress Adaptation by Sinorhizobium meliloti Rm1021
Source: PLoS One. 2013 Mar 13;8(3):e58028. doi: 10.1371/journal.pone.0058028 (PMC3596328; doi:10.1371/journal.pone.0058028)
Supplement: Table S1 — Gene-specific primers used for qPCR. (DOCX) [file pone.0058028.s001.docx]

Table S1. Gene-specific primers used for qPCR

| Gene | Function | Forward primer | Reverse primer |
| --- | --- | --- | --- |
| SMc02641, *rkpK* | UDP-glucose 6-dehydrogenase | CTTGCGGTTGTCGTTGACG | TTCATCAACGAGATTGCCGA |
| SMc01949, *livG* | High-affinity branched-chain amino acid transport ATP-binding ABC transporter | CTGCATCACCGGTTTCTACA | ATGCGGATGTTCTGGAAGGT |
| Smc01513, *hmuS* | Hemin transport protein | AGCCTGCAGTTCTTCGACAA | TCCAGGCGAAGATCTTCGAC |
| SMc02085,*exbB* | Biopolymer transport transmembrane protein | TCTTCTCTGCTCACGCGTAT | AATTCATGATTCCCCAGACG |
| SMc20286 | Putative opine dehydrogenase | CTTCGGTCTGTCGGTCAGAA | TGCATCTGCTGGTTCATGTC |
| SMb20282 | Spermidine/putrescine ABC transporter, permease component | CATGAGCTTCAAGGATGCGA | AACTGCTTGTCGTTGAGCAG |
| SMa1220,  *fixN* | FixN_1_ cytochrome *c* oxidase subunit 1 | GGAATGGTACGTCGACCTCT | CACGTAGATGTGCGGTTCTT |
| SMc01659 | ABC transporter, periplasmic component | CTCTCCTTCGAGACCATCCT | CGGTGCTTTCGAGATATTCC |
